# Impact of contrasting poultry exposures on human, poultry, and wastewater antibiotic resistomes in Bangladesh

**DOI:** 10.1128/spectrum.01763-23

**Published:** 2023-11-16

**Authors:** Alexander D. Williams, Emily Rousham, Andrew L. Neal, Mohammed Badrul Amin, Jon L. Hobman, Dov Stekel, Mohammad Aminul Islam

**Affiliations:** 1 Laboratory of Data Discovery for Health Ltd, Hong Kong Science and Technology Park, Tai Po, Hong Kong; 2 School of Public Health, University of Hong Kong, Pok Fu Lam, Hong Kong; 3 Centre for Global Health and Human Development, School of Sport, Exercise and Health Sciences, Loughborough University, Loughborough, United Kingdom; 4 Net-Zero and Resilient Farming, Rothamsted Research, North Wyke, United Kingdom; 5 Laboratory of Food Safety and One Health, Laboratory Sciences and Services Division, icddr,b, Dhaka, Bangladesh; 6 School of Biosciences, University of Nottingham, Sutton Bonington Campus, Sutton Bonington, Leicestershire, United Kingdom; 7 Department of Mathematics and Applied Mathematics, University of Johannesburg, Auckland Park, South Africa; 8 Paul G. Allen School for Global Health, Washington State University, Pullman, Washington, USA; Chung-Ang University, Anseong, Gyeonggi-do, South Korea

**Keywords:** antibiotic resistance, metagenomics, one health, environmental microbiology, occupational exposure

## Abstract

Interactions between humans, animals, and the environment are considered critical foci for addressing antimicrobial resistance (AMR). However, One Health data on AMR in low- and middle-income countries are presently scarce. Using metagenomics, we investigated whether and how the fecal antibiotic resistomes of humans are influenced by exposure to intensive and non-intensively reared poultry within contrasting settings of urban wet markets (*n* = 13) and rural households (*n* = 7) in Bangladesh. We also considered poultry (*n* = 10) and wastewater (*n* = 10) resistomes in these settings. We found that occupational poultry exposures did not significantly alter the human fecal resistome. In contrast, macrolide-lincosamide-streptogramin and streptothricin antibiotic resistance genes (ARGs) were enriched in poultry from urban wet markets relative to rural household chickens. Wastewater had the highest ARG richness, though this was only partially explained by poultry cecal and human fecal sources. Wastewater also contained clinically significant carbapenem ARGs. This study therefore provides critical insight into the distribution of ARGs in Bangladesh.

**IMPORTANCE:**

Through the use of DNA sequencing, our study shows that there is no significant difference in the antibiotic resistance genes found in stool samples taken from individuals with high exposure to poultry routinely fed antibiotics and those without such exposure. This finding is significant as it suggests limited transmission of antibiotic resistance genes between poultry and humans in these circumstances. However, our research also demonstrates that commercially reared poultry are more likely to possess resistance genes to antibiotics commonly administered on medium-sized farms. Additionally, our study highlights the under-explored potential of wastewater as a source of various antibiotic resistance genes, some of which are clinically relevant.

## INTRODUCTION

The fecal carriage rate of extended-spectrum beta-lactamase producing Enterobacterales (ESBL-E) has been increasing globally with the highest prevalence rates in South Asian countries (1). Intestinal colonization with antibiotic-resistant organisms in humans poses an elevated risk of subsequent infection with resistant organisms. Besides, colonized humans and animals shed ESBL-E through feces which are often disposed into the environment due to poor sanitation infrastructure. Therefore, reduction of community carriage of antimicrobial resistance (AMR) has been considered as a major step in combating AMR (2). There are many drivers for drug-resistant infections in low- and middle-income countries (LMICs), including unregulated sales of antibiotics, misuse of antibiotics in clinical medicine and agriculture, poor sanitation and sewerage infrastructure, and overall poor governance in health care.

Unregulated use of antibiotics in intensive farming of food-producing animals and in aquaculture has become a common practice in many LMICs (3). More alarmingly, antibiotics critical for human health are often used in animal and fish farming, resulting in development of resistance to clinically important antibiotics among bacterial pathogens of concern for human health (4). It has been suggested that transmission from animals to humans of bacteria and/or mobile genetic elements carrying ESBL-encoding genes may contribute to human infection with ESBL-producing *Escherichia coli* (ESBL-Ec); however, existing evidence suggests this occurs infrequently (5, 6). Nonetheless, in a community-based survey in Bangladesh, we found that 67.5% of healthy adults and 68.0% of poultry were colonized with ESBL-Ec while 92.5% of wastewater samples tested positive for ESBL-Ec with similar prevalence rates in rural and urban settings (7).

Previous research among small-scale commercial broiler farms in Bangladesh [small- (300–2,000 birds) to medium (2,000–5,000 birds)-sized family-run businesses] found multiple antibiotics were added routinely to water or poultry feed as prophylaxis and for growth promotion (8). The close proximity of humans and animals in these farms provide opportunities for bidirectional transmission of antibiotic-resistant bacteria (ARB) and antibiotic-resistant genes (ARGs) between hosts. Moreover, the lack of sanitation infrastructure, waste management, and waste treatment in both rural and urban areas has led to widespread environmental contamination by fecal bacteria, resistance genes, and antibiotic residues. Small-scale commercial poultry farmers and the sellers of live poultry in urban markets in Bangladesh also face direct exposure to animal tissues, waste products, and associated ARB and ARGs because they use little or no protective clothing, gloves, or masks (9).

The contribution of different sources to AMR in the environment is considered an important gap in current understanding (10). Although ARB have been shown to be prevalent in humans, animals, and environmental samples in Bangladesh, the diversity and abundance of ARGs and the extent of sharing between different hosts and environmental compartments are less studied.

Only a few studies in LMICs have assessed all three domains (human, animal, and environment) of the One Health paradigm for AMR surveillance (11). Importantly, there is a lack of data on the sharing of microbiome and antibiotic resistomes between humans, domestic/farmed animals, and environments within similar ecological units in LMIC settings, where humans and livestock often live in close proximity, and sanitation is often inadequate. Metagenomics-based surveillance makes it possible to compare resistomes and bacterial population structures within and between different ecological settings. Organisms that are significant contributors of ARGs within a population, including culturable, non-culturable and under-studied organisms, can be detected by metagenomic analysis. Recently sewage and wastewater surveillance for AMR using metagenomics has gained traction due to its advantages over traditional population-based surveillance, which is resource intensive for many LMICs (12, 13). Paradoxically, many LMIC settings that are considered as hotspots for AMR have the sparsest data on metagenomic-based AMR surveillance.

In this study, we aimed to understand the dynamics of AMR transmission in Bangladesh using shotgun metagenomic profiling. Specifically, we aimed to determine whether the human gut resistome can be explained by exposure to poultry and wastewater in two contrasting settings of (i) urban wet markets selling commercial broilers produced by small-scale farmers with intensive use of antibiotics and large flocks (intensive) and (ii) households in rural villages with small numbers of free-range backyard poultry raised with no antibiotics or antibiotics used for treatment purposes only (non-intensive). In each of the two settings, we systematically collected samples from humans, poultry, and the surrounding wastewaters as part of a purposefully designed One Health AMR study (14). In urban wet markets and rural households, we collected human samples from those with and without direct occupational contact with poultry to determine whether the resistome varied according to poultry exposure. Additionally, we compared the composition of the poultry gut resistome in urban wet market broilers versus domestic backyard poultry. We also investigated their contributions to downstream contamination through direct disposal of wastewater.

We address our main research objectives as follows: to establish the influence of poultry exposure and production setting on the poultry gut, human fecal, and wastewater resistome, we assessed both the relative abundance and diversity of ARG compositions. Sample group multivariate dispersions were compared to further test within- and between-group differences. Assembled contigs were examined to characterize the genetic context of ARGs including their potential mobility and taxonomic association with bacterial pathogens of clinical concern. Resistome overlap and a source-tracking algorithm were used to assess the extent of resistome sharing.

## MATERIALS AND METHODS

### Sampling strategy

The samples discussed in the present work were collected as part of a One Health surveillance study on ESBL-Ec in Bangladesh (7, 11). Human fecal, poultry cecal, and wastewater samples were collected between February and October 2017, from urban wet markets in Dhaka city and from households in a rural area in the Tangail district. Wet markets in Dhaka sold live broilers which were slaughtered, defeathered, and processed on site at the request of customers. Solid waste from poultry processing was disposed into general municipal waste sites at the markets while liquid waste including poultry blood and feces was disposed directly into wastewater drains (9). Wastewater in urban market outlets typically contain waste from all parts of the market including large and small animals, fish, and fresh produce. The broilers sold in wet markets were sourced from commercial farmers and sold within 24–48 hours of arrival. Antibiotics are not typically administered to birds by wet market stall holders due to short stocking periods. Antibiotic usage records were not available for the specific birds sampled. Evidence from small-scale commercial farms in the wider study who supplied to Dhaka markets, however, revealed that 95% of farmers (out of 40 farms surveyed) fed antibiotics to their broilers throughout the production cycle with the most commonly used antibiotics being tetracycline, fluoroquinolones (ciprofloxacin and enrofloxacin), and macrolides (erythromycin or tylosin) (7). Some farms also administered colistin sulphate. Eighty percent of farms administered multiple antibiotics contemporaneously (7). Backyard poultry in rural households typically roamed freely in courtyards or buildings and frequently entered food preparation areas. These poultry were fed kitchen scraps and scavenged food (9). The participating rural households owned between 4 and 20 chickens. Wastewater runoff from rural households went into either household ponds, drainage ditches, or other surface waters (15).

In each setting (rural households vs wet markets), we selected humans with and without daily occupational exposure to poultry, these were considered as “high-” and “low-” poultry exposure groups. In the rural households, this comprised of participants from households with backyard poultry (high exposure) versus households without poultry (low exposure). In the urban wet markets, this comprised of market stall holders selling and slaughtering live broilers (high exposure) versus market stall holders selling general groceries (low exposure).

The current work focuses on a subset of 40 samples which were processed for metagenomic sequencing. This included 20 human fecal samples with high (*n* = 8) versus low poultry exposure (*n* = 12) (rural householders with backyard poultry, *n* = 2; rural households without backyard poultry, *n* = 5; poultry slaughterers in urban wet markets, *n* = 6; and grocery stall holders in urban wet markets, *n* = 7), 10 poultry cecal samples (backyard poultry from rural households, *n* = 4; broilers from urban wet markets, *n* = 6) and 10 wastewater samples (rural households, *n* = 4; urban wet markets, *n* = 6). Wastewater samples were collected from the outlet of the main wastewater drain at each urban market and rural household. More detailed metadata are available in Supplementary Table S1 . We aimed to analyze shared and distinct resistomes according to sample origin (human, poultry, or wastewater), occupational exposure to poultry, and setting (urban wet markets versus rural households).

### Sample collection

Human fecal samples were provided by study participants using a sterile stool sample container supplied by field staff. All fecal samples were stored on ice within 2 hours of collection. For poultry ceca samples, chickens were slaughtered, and the skin was removed on site by the owner following their usual procedures. The carcass was placed in a sterile bag, sealed immediately and placed in a cool box on ice for transportation to the laboratory. Wastewater samples were collected by taking approximately 150 mL of wastewater from three locations along the runoff drain adjacent to the selected household or market, by dipping a sterile container into the drain. Wastewater samples were then pooled by location in a sterile 500-mL plastic bottle (Nalgene, New York, USA) and placed on ice for transportation.

All samples were transported to the laboratory within 5 hours of collection maintaining the cold chain, refrigerated on arrival, and processed within 18 hours of collection. In the laboratory, ceca samples were taken from the chicken carcass aseptically by cutting the keel bone, identifying, and excising the cecum with sterilized scissors, and extracting the cecum contents.

### Ethical considerations

Written and verbal information about the study was provided, and participating volunteers gave written informed consent. Ethical clearance was obtained from icddr,b (International Centre for Diarrhoeal Disease Research, Bangladesh) (PR-16071) and Loughborough University, UK (R17-P037). Further details of the wider study including protocols, questionnaires, and data sets are available at https://catalogue.ceh.ac.uk/documents/279c2e56-24fe-41d2-ab4c-eda1c1e8eae0. Local authorities were informed prior to wastewater sampling in markets. Broiler poultry and backyard chickens were purchased on a commercial basis and slaughtered by the owner/vendor who had consented to participate. Owners followed their normal procedure for slaughtering animals as for domestic consumption or commercial sale.

### Metagenomic sequencing and sequence cleaning

DNA was extracted from wastewater samples using the MO Bio Power Water DNA Isolation Kit (MO BIO Laboratories Inc., Carlsbad, CA, USA); the QIAamp DNA Stool Mini Kit (Qiagen, UK) was used for human fecal and poultry cecal samples. Briefly, 100 mL of wastewater was passed through 0.22-µm cellulose membrane filters and DNA was extracted directly from the filter concentrate using the MO Bio Power Water DNA Isolation Kit following the manufacturer’s protocol. In the case of human fecal and poultry cecal samples, DNA was extracted from 0.25 g of fecal/cecal content using QIAamp DNA Stool Mini Kit (Qiagen) following the manufacturer’s protocol. DNA purity and concentration were assessed with a NanoDrop 2000 spectrophotometer (Thermo Scientific) and a Qubit 3.0 fluorometer (Life Technologies), respectively. DNA fragmentation was checked by running 2 µL of extracted DNA on 1% agarose gel. All DNA samples were stored at −80°C. Short read metagenomic sequencing (Illumina NovaSeq 6000, 150-bp paired-end libraries) of extracted DNA was carried out by Novogene (Novogene Co. Ltd., Cambridge, UK).

Removal of sequencing adapters and quality trimming was carried out with *Trimmomatic* v0.38 (16) (settings: 2:30:10, leading:3, trailing:3, slidingwindow:4:15, and minlen:36). Host and other non-bacterial reads were removed by mapping with *bowtie2* v2.3.5 (17) using default settings. The read removal strategy was based on the biological context of samples. For human stool samples, the human genome (RefSeq GCF_000001405.40_GRCh38.p14) was used for reference mapping and read exclusion. For poultry ceca, the broiler chicken (*Gallus gallus*) genome was used for reference mapping (RefSeq GCF_016699485.2_bGalGal.mat.broiler.GRCg7b). Finally, for wastewater samples, human, broiler chicken and cattle (*Bos taurus*; RefSeq GCF_002263795.2_ARS-UCD1.3) genomes were used for reference mapping.

### Metagenome assembly

Following quality control and non-target sequence removal, samples were assembled individually using *Megahit* v1.2.9 with 21, 29, 39, 59, 79, 99, 119, and 141 *k*-mer intervals (18).

### Annotation of antimicrobial resistance genes in unassembled data and contigs

ARGs were annotated with a locally installed copy of *ARG-OAP* v2.0 (19) using an ARG sequence identity cut-off of 80% and minimum query alignment length > 25 amino acids, expect-value 1 × 10^−7^ (20 – 22). Normalization of ARGs by estimated genome number was carried out by *ARG-OAP* which uses *diamond* v2.0.15 (23) to identify a suite of 30 universal single-copy genes.

Contigs were screened for ARGs based on protein homology using the RGI online platform (resistance gene identifier, v6.0.0), combined with the CARD database (v3.2.5) (24). Only matches exceeding 90% identity and coverage of reference protein sequences were considered in analyses.

### Taxonomic classification of unassembled data and resistance gene-bearing contigs

After quality checking, taxonomic classification of short reads was carried out with *Kaiju* v1.7.1 (25) in combination with the pre-built *nr_euk* protein database (downloaded from *Kaiju* webserver March, 2019). The database contains non-redundant protein sequences for Archaea, Bacteria, Viruses, fungi, and microbial eukaryotes from the NCBI-BLAST database (26). *Kaiju* was run in “greedy” mode, allowing three mismatches.

For ARG-bearing contigs of particular interest, NCBI megaBLAST (27) was performed to assign putative taxonomy.

### Data exploration and statistical analysis

To evaluate whether the predicted resistome composition varied between sample origin (poultry cecal, human fecal, and wastewater) and setting (rural households and urban wet markets), principal coordinates analysis (PCoA) was first used to generate unconstrained ordinations of sample composition (Hellinger distances of bacterial cell normalized ARG category and subtype abundance). As PCoA indicated clustering, permutational multivariate analysis of variance (PERMANOVA) was used to establish if significant multivariate differences were observed between groups, following testing for heterogeneity of multivariate dispersion using the PERMDISP test. We performed discriminant analysis using canonical analysis of principal coordinates (CAP) in Hellinger space. To avoid model over-parameterization, we identified the optimal PCoA axes to employ in CAP by maximizing a leave-one-out allocation success to treatments. Having established any clustering within the multivariate ordination, we determined the likely ARG categories associated with sample clustering using Pearson correlation coefficients to determine linear relationships between ARGs and clusters. PCoA, PERMDISP, PERMANOVA, and CAP were all conducted using the *PERMANOVA+* add on to *PRIMER* version 7.0.20 (28, 29). For all tests, probabilities were based upon 99,999 permutations (denoted *P*_perm_). In cases where the number of observations was insufficient to allow at least 999 permutations for post hoc pairwise tests, Monte Carlo probabilities (denoted *P*_MC_) were calculated based upon an asymptotic permutation distribution.

**Fig 1:**
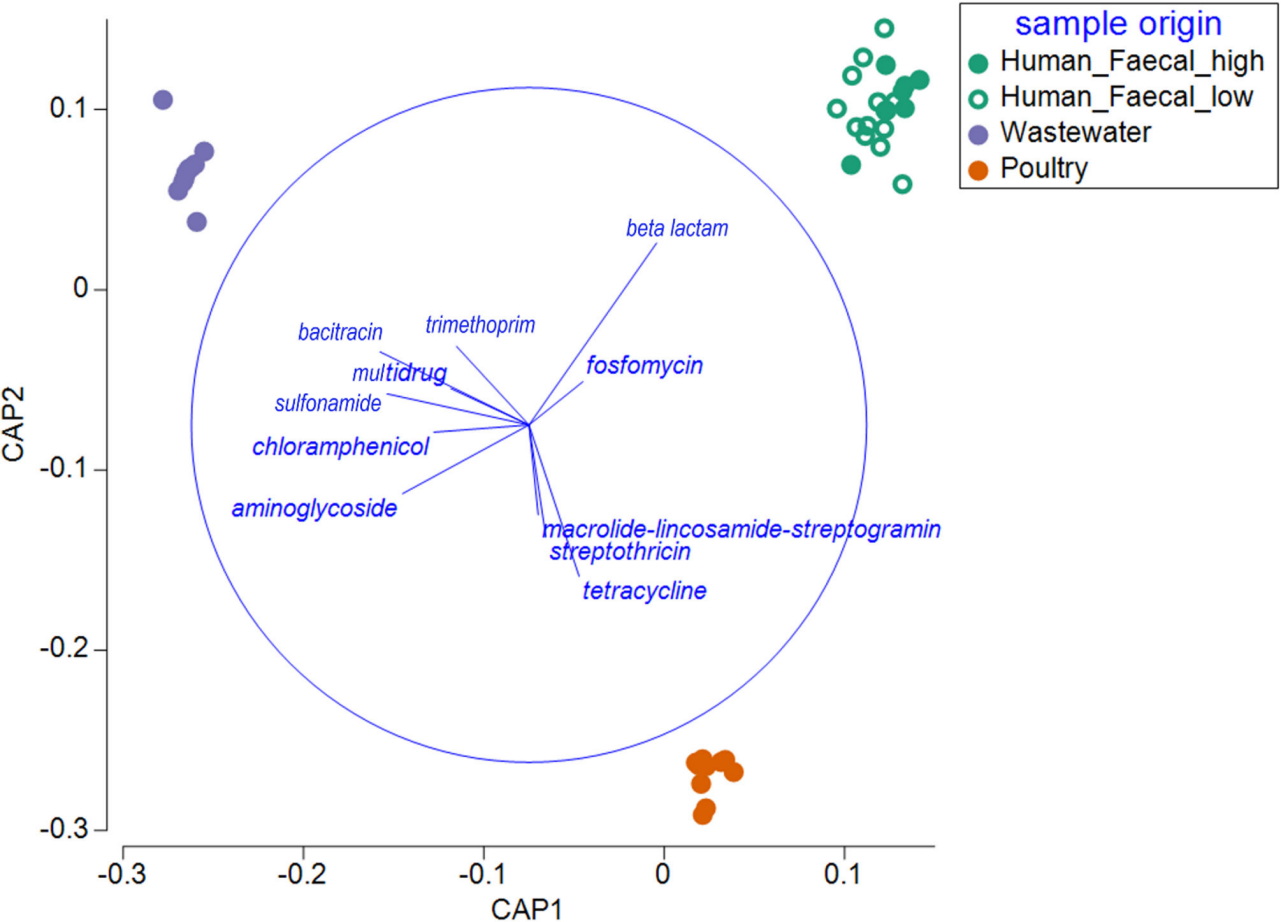
ARG category-contingent CAP identifies significant separation of ARG assemblages between sample origins (trace statistic = 2.5, *p*_perm_ = 1×10^−5^). CAP1-squared canonical correlation (δ²) = 0.997, CAP2 δ² = 0.988. Cross-validation based upon a leave-one-out allocation of observations to groups was associated with a 17.5% mis-classification error rate, resulting from mis-classifying human fecal samples with high and low poultry exposure. Vector overlays represent multiple partial correlations between CAP axes and ARG categories (*r* > 0.2). The circle has a radius of *r* = 1; the relative size and position of origin are arbitrary with respect to the underlying plot. Vector length and direction indicate the strength and sign, respectively, of the association between each ARG category and the CAP axes.

Based on PERMANOVA, univariate differential abundance of ARGs in rural and urban settings was only assessed in poultry cecal samples. Only ARG categories associated with poultry ceca having *r* > 0.2 were analyzed, see vectors associated with CAP analysis (Fig. 1). To account for non-normal data distribution and heterogeneity of variance between sample data, Welch’s unequal variances *t*-tests were combined with 99,999 Monte Carlo permutations to determine probabilities associated with *t*. The Benjamini-Hochberg procedure for false discovery rate adjustment was applied to the resulting probabilities (denoted *P*_adj._).

ARG subtype diversity was assessed using Hill numbers implemented by the *iNEXT* v3.0.0 (30) package in R with 40 knots and confidence intervals bootstrapped 399 times.

Other exploratory visualizations included boxplots of genome-normalized abundances of the five most abundant ARG subtypes by sample origin and ARG categories associated with sample origin according to CAP (*r* > 0.2). Abundant phyla and genera were summarized by sample origin in compositional plots produced with the R package *microbiome v1.18.0* (31) (Supplementary Fig. S1).

Further investigation into associations between specific ARG subtypes, taxa, and sample origins was guided by contigs of interest and initial data exploration. In doing so, we sought to avoid data dredging. We focused on the following objectives: (i) identifying whether specific taxa could be shown to drive dominant features of the resistome in the three sample origins (human, poultry, and wastewater), (ii) identifying taxa associated with multi-drug resistance determinants (three or more different antibiotic categories), and (iii) determining the distribution of the WHO priority one antibiotic-resistant bacteria of critical concern (32). Where appropriate, associations between the centered log ratio (CLR) of taxa and ARG count data were tested using major axis regressions in R using *lmodel2* v1.7–3 (33). Only contigs > 1 kbp were used for assigning putative taxonomy.

Antibiotic resistome source contribution analysis was carried out with *FEAST* algorithm R package (34, 35) where poultry, human, and wastewater samples were collected from the same urban wet market site (*n* = 3). *FEAST* was supplied count data for the ARG subtype and was run using default settings (expectation–maximization iterations = 1,000, coverage = minimal sequencing depth in sink and sources).

## RESULTS

### Characteristics of sequencing data

On average, sequencing yielded approximately 11 GB of data per sample. A total of 15.4M contigs were generated from the metagenomic assembly of 40 samples with a range of 200 bp to 782.9 kbp in length. Of these, 1,662 contigs contained at least one ARG and 1,553 were ≥1 kbp which were selected for taxonomic profiling (poultry, *n* = 363; human feces, *n* = 640; and wastewater, *n* = 550). Two samples (DL_164_WW2 and DL_087_WW2) were shown to be heavily contaminated (>80% reads mapped concordantly) with broiler chicken genetic material. Both samples originated from urban wet markets; the high proportion of reads mapping to broiler chicken is therefore likely to be a consequence of poultry slaughtering and waste disposal practice.

### Sample origin has the greatest impact on resistome composition and setting has a secondary influence on poultry and wastewater ARG carriage

Around 15% of core ARG subtypes (here defined as subtypes detected in at ≥2 samples in one or more sample origin; total *n* = 1,047) were shared by all sample origins (Supplementary Table S2). However, analyses show that the overall resistome compositions associated with each sample origin remained distinct. This is evident in the separation of poultry cecal, human fecal, and wastewater samples shown by unconstrained PCoA and CAP of ARG subtype and category data (Fig. 1; see Supplementary Fig. S2 and Fig. S3 for PCoA).

**Fig 2:**
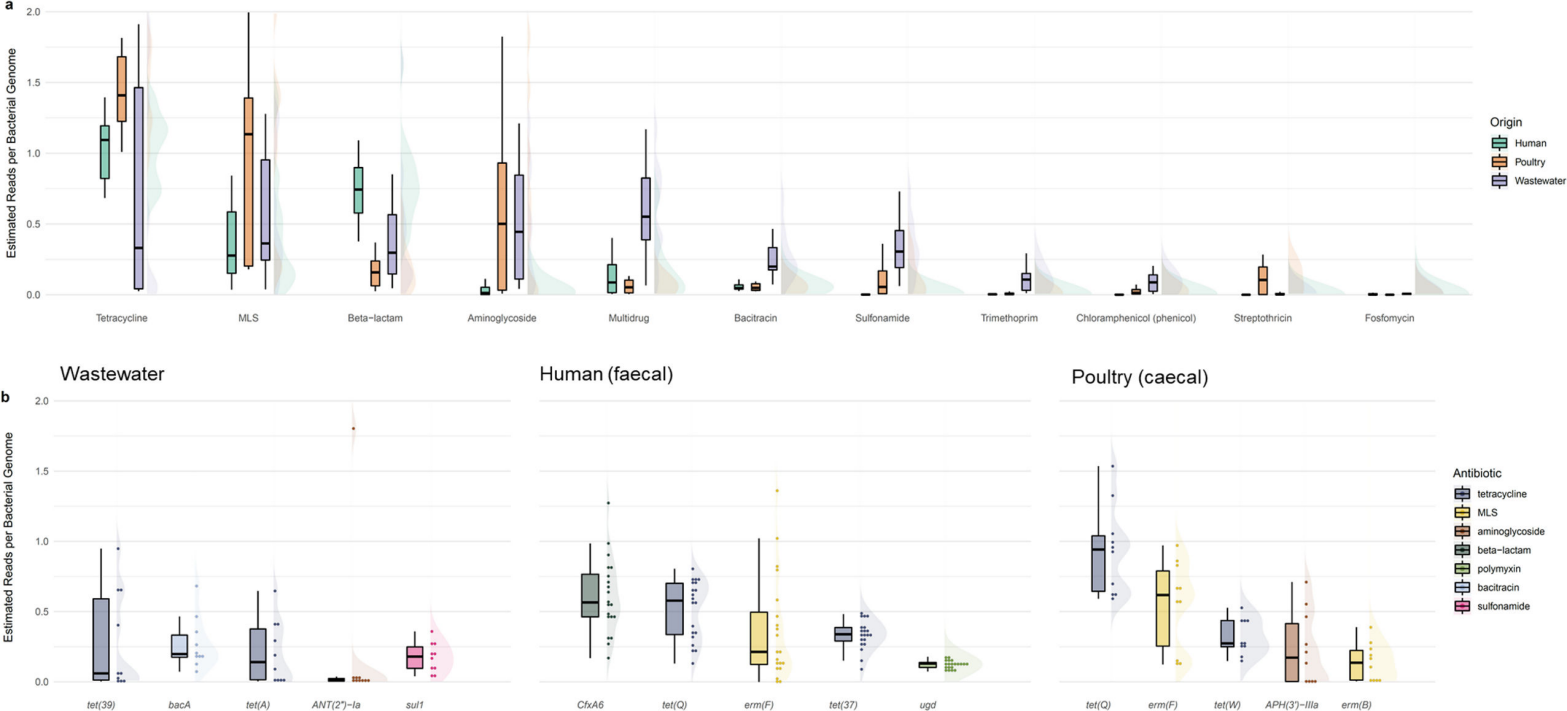
Summary of abundant antibiotic resistance genes in each sample origin. (a) Boxplots and half-violin plots of estimated ARG copies per genome for ARG categories highlighted by canonical analysis of principal coordinates (*r* > 0.2) (compared by sample source); (**b**) Boxplots and half-violin plots with stacked dots showing the five most abundant ARG subtypes by mean in each sample source (wastewater, human fecal, and poultry cecal), ordered by mean estimated reads per bacterial genome.

Genes associated with tetracycline resistance were the most abundant ARG category on average, regardless of sample origin [mean abundance, 1.2 copies per bacterial genome (cpbg), ±0.12 standard error of the mean] (Fig. 2A). Based on abundance data, poultry ceca samples were the most enriched with tetracycline resistance genes (1.79 ± 0.18 cpbg) compared with both wastewater (1.07 ± 0.32 cpbg) and human fecal samples (1.05 ± 0.05 cpbg) (Fig. 2A). The next most abundant ARG categories overall were macrolide-lincosamide-streptogramin (MLS) resistance genes (0.63 ± 0.09 cpbg) and beta-lactam resistance genes (0.55 ± 0.06 cpbg); these were most associated with poultry cecal (1.10 ± 0.22 cpbg) and human fecal samples (0.80 ± 0.06 cpbg), respectively (Fig. 2A).

At the subgroup level, no single ARG was dominant across sample groups. On average, the tetracycline ARG *tet*(*Q*) was the most prevalent subtype in poultry samples and *tet*(*Q*) and the beta-lactam resistance subtype *cfxA6* in human fecal samples (0.51 ± 0.05 and 0.61 ± 0.06 cpbg, respectively), whereas wastewater samples were marked by greater between-sample variability, and therefore, multiple similarly abundant subtypes were present (Fig. 2B).

### Resistome diversity

ARG subtype diversity was assessed using Hill numbers. There was a significant difference in median ARG subtype richness (^0^ *D*) between sample origins (human, poultry, and wastewater) (Kruskal-Wallis test; *χ*² = 23.3, two-tailed df = 3, *P* = 3 × 10^−5^), but not setting (urban wet market versus rural households) (Kruskal-Wallis test; *χ*² = 1.5, df = 1, *P* = 0.214) (Supplementary Fig. S4). Post hoc pairwise comparisons of median ^0^ *D* indicated significant differences between wastewater (median ^0^ *D* = 526) and all other sample origins (smallest difference; wastewater and poultry cecum, median ^0^ *D* = 223, Mann-Whitney *Z_U_* = 3.6, *P*_adj._ = 0.00074). No significant differences in richness estimates were observed between poultry cecum or human fecal samples (median ^0^ *D* of human feces from high or low poultry exposure were 137 and 171, respectively). However, a significant interaction was identified between setting and origin (Kruskal-Wallis test; *χ*² = 27.5, df = 5, *P* = 4.573e^−5^). Despite this, group-wise testing indicated no significant differences after correction for multiple testing (Kruskal-Wallis tests: poultry cecal; *χ*² = 6.55, df = 1, *P*_adj._ = 0.06312; human fecal; *χ*² = 0.01, df = 1, *P*_adj._ = 1.00; and wastewater; *χ*² = 0.73, df = 1, *P*_adj._ = 1.00).

### Multivariate analyses of ARG compositions

For the multivariate test of ARG assemblages, no significant heterogeneity of multivariate dispersion was observed for either sample origin or setting for ARG type. For the ARG subtype, however, there was significant heterogeneity of multivariate dispersion associated with sample origin (PERMDISP, pseudo-*F* = 5.9, *P*_perm_ = 0.0058). For the ARG subtype, dispersion was greater for wastewater samples than either human fecal or poultry cecal samples (wastewater relative to human fecal, high exposure: *t* = 4.1, *P*_perm_ *=* 0.003; wastewater relative to human fecal, low exposure: *t* = 4.6, *P*_perm_ = 0.0004; and wastewater relative to poultry samples: *t* = 3.5398, *P*_perm_ = 0.003). Testing the effect of sample setting (rural households, urban wet markets) and sample origin (human fecal, poultry cecal, and wastewater) indicated both sample origin (PERMANOVA, pseudo-*F* = 24.8, *P*_perm_ = 1 × 10^−5^) and setting (pseudo-*F* = 3.7, *P*_perm_ = 0.0087) exerted a significant influence upon ARG subtype assemblages. Significant interaction between the two factors was also identified (pseudo-*F* = 3.0, *P*_perm_ = 0.001). Post hoc pairwise comparisons indicated that while there was no significant difference between ARG subtype assemblages in fecal samples collected from human subjects with either high or low exposure to poultry, assemblages in poultry ceca, human feces, and wastewater were all significantly different; however, we cannot discount the fact that this may reflect differences in dispersion between the different environments. This pattern was the same for both rural households and urban wet markets. In addition, differences in ARG subtype assemblages were also observed between wastewater (*t* = 1.9, Benjamini-Hochberg adjusted *P*_MC_ = 0.014) and poultry ceca (*t* = 3.3, Benjamini-Hochberg adjusted *P*_MC_ = 0.0007) from rural households and urban wet markets.

### Setting influences total normalized ARG abundance

After log_10_ transformation, total normalized ARG abundance varied significantly between settings (Welch *t* = −4.17, df = 29.623, *P* = 0.00024), with urban wet market samples exhibiting higher mean total ARG abundance than rural households. Due to small sample size, unequal variance, and non-normal distributions which could not be resolved by transformation, we did not carry out further statistical tests on interaction effects between sample origin and setting. However, we observed trends which suggest the difference between urban wet market and rural households is primarily driven by wastewater samples (Supplementary Fig. S5).

### Wastewater resistomes are a sink for multiple, unsampled sources

*FEAST* source attribution where wastewater, human fecal, and poultry cecal samples were collected from the same urban wet markets showed that the majority of the wastewater resistome could not be explained by either potential source (mean 75.87% ± 4.50 unknown source). In urban wet markets, the poultry resistome made a greater contribution to corresponding wastewater streams (21.39% ± 3.08) than human fecal sources (2.73% ± 0.01%).

### MLS and streptothricin ARGs are enriched in broiler poultry ceca

**Fig 3:**
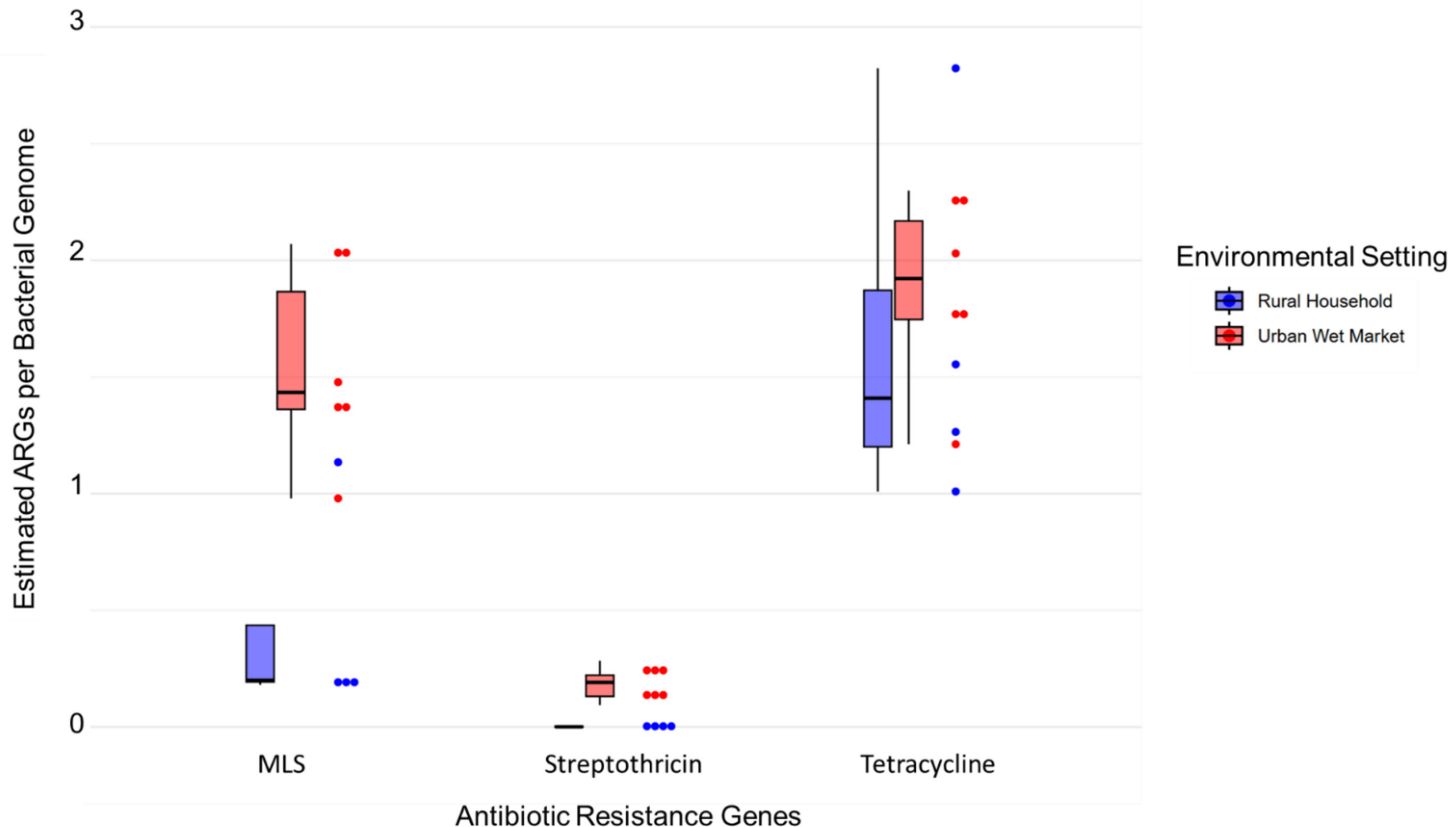
Differences in the abundance of select antibiotic resistance gene categories in poultry ceca samples collected from urban wet market and rural household chickens. Boxplots and stacked dot plots of MLS, streptothricin, and tetracycline resistance gene abundance are shown.

The multivariate differences between rural backyard chickens and urban wet market broiler poultry resistomes indicated by PERMANOVA were further investigated. Of the three ARG categories shown to associate with poultry resistomes according to CAP vector overlay (Fig. 1), MLS and streptothricin resistance genes were significantly enriched in urban broilers (Welch *t* = 3.8, *P*_adj._ = 0.0145; Welch *t* = 6.3, *P*_adj._ = 0.0245, respectively; Fig. 3). However, tetracycline resistance genes were prevalent at similar levels in urban and rural bird ceca (Welch *t* = 0.51, *P*_adj._ = 0.567). Given the small sample size and numerous ARG subtypes, statistical tests are not reported; nonetheless, heatmaps of MLS and streptothricin ARG subtypes are provided for poultry samples in Supplementary Figure S6. Several ARG categories were shown to be indicative of wastewater resistomes; however, differences between urban and rural samples were not tested due to low statistical power.

### Urban and rural wastewaters contain ESBL and carbapenem resistance genes

According to the WHO Global Priority Pathogens List, priority one “critical concern” antibiotic-resistant pathogens include ESBL-E, carbapenem-resistant *Enterobacteriaceae* (CRE), *Acinetobacter baumannii*, and *Pseudomonas aeruginosa*. While 21 contigs > 1 kbp contained ESBL/carbapenem resistance genes, after cross-validation of taxonomy, only three contigs unambiguously fulfilled the “critical concern” criteria; all originated from wastewater samples (Supplementary Table S3).

Putative *Enterobacteriaceae* contigs containing TEM family genes were identified in human fecal (high and low exposure) (*n* = 2), poultry (*n* = 3), and wastewater (*n* = 3) contig libraries; however, all were classified as broad-spectrum beta lactamases (BSBLs) TEM-1. Similarly, SHV genes in wastewater (SHV-27, *n* = 1; SHV-110, *n* = 1) and human fecal (SHV-1, *n* = 1; SHV-11, *n* = 2; and SHV-61, *n* = 1) contigs were primarily non-ESBL variants. PER and RSA group ESBL genes were detected; however, they could not be confidently associated with *Enterobacteriaceae*.

OXA group genes were the most frequently identified beta-lactamases on contigs across the entire data set (*n* = 50, *n* = 49 > 1 kb) and were found in all sample origins. However, OXA ESBL/carbapenemase genes could not be definitively associated with *Enterobacteriaceae*. For example, one rural wastewater contig carried an OXA-372 family carbapenemase gene (36) (OXA-641-like); OXA-641 is known to be found in *Morganella* spp (Supplementary Fig. S7a). Additionally, one chromosomal *Acinetobacter baumannii* rural wastewater contig encoded a variant (OXA-65) belonging to the OXA-51 family (37) (Supplementary Fig. S7b). Another *Acinetobacter* spp. contig derived from urban wastewater contained OXA-58, also a potential carbapenemase (38) (Supplementary Fig. S7c).

VEB group ESBLs were also identified; these were only recovered from wastewater contigs and could not be categorically associated with *Enterobacteriaceae*. However, the longest VEB-bearing contig (7.7 kbp) contained a class 1 integron cassette with the potential to confer multidrug resistance across four different antibiotic categories (beta-lactam, phenicol, aminoglycoside, and fluoroquinolone) (Supplementary Fig. 7d). NCBI-BLAST indicated this contig shared high nucleotide homology with enterobacterial plasmids [*Klebsiella quasipneumoniae*, 99.69% identity, 100% query coverage (CP058135.1), and *Escherichia coli*, 99.98%, 100% query coverage (LC745731.1)] and non-enterobacterial chromosomes [*Aeromonas veronii*, 100% identity, 94% query coverage (CP054855.1)]. Finally, three wastewater contigs contained genes from the GES carbapenemase family. Urban and rural wastewater contigs (1.3–1.5 kbp) containing *bla*_GES-2_ shared high homology with *Pseudomonas aeruginosa* chromosomes and enterobacterial plasmids (Supplementary Fig. 7e). Another urban wastewater contig (1.8 kbp) contained *bla*_GES-5_ with nucleotide homology among Gammaproteobacteria, including chromosomal sequences of *Pseudomonas aeruginosa* [99.90% identity, 70% query coverage (KY860573.1)] and *Klebsiella pneumoniae*-associated plasmids [100% identity, 70% query coverage (MN436715.1)] (Supplementary Fig. S7f, Supplementary Table S3).

### Select ARGs are associated with specific genera and sample origin, while most are widely dispersed

To investigate whether select ARGs are linked to specific bacterial hosts and sample origin, contigs were used to direct correlation analyses between short-read abundance data of ARGs and taxa. Since the prevalence of beta-lactam resistance genes were shown to distinguish human fecal samples from those collected from poultry ceca and wastewater (see Fig. 1 and 2B), dominant beta-lactam ARGs were identified. The principal beta-lactam ARG in human samples was *cfxA6*. Contig data suggested CFXA family genes were typically associated with *Bacteroides* and *Prevotella* spp. We found a significant positive correlation between the CLR of *Prevotella* spp. and *cfxA6* counts across the entire data set (*R* = 0.95, *t* = 18.954, *P* = 5.799111e^−21^) and positive trends held within each sample origin. However, correlations with *Bacteroides* spp. or phylum Bacteroidetes were inconsistent within samples of different origin. Although contig analyses show *cfxA* genes were present in both *Bacteroides* spp. and *Prevotella* spp., these findings indicate *Prevotella* spp. were the most consistent carrier of *cfxA6* in these data. Correspondingly, *Prevotella* spp. accounted for over half of all reads in the majority (16/20) of human fecal samples based on taxonomic assignment with Kaiju, whereas the same genus was comparatively rare in both poultry fecal and wastewater samples.

In contrast, dominant ARGs distinguishing wastewater and poultry samples could not be robustly associated with specific taxa, indicating the dispersal of these genes across distantly related taxa via mobile genetic elements including class 1 integrons as shown by contig data.

## DISCUSSION

In this study, we aimed to explore the sharing of antimicrobial resistomes between humans, poultry, and wastewater, with and without exposure to intensive poultry production. We also contextualized the composition of the poultry gut resistome in relation to production setting and its contribution to environmental contamination via wastewater outlets.

We found that antimicrobial resistomes were largely characterized by sample origin (human feces, poultry ceca, or wastewater), although those of poultry ceca and wastewater were additionally influenced by setting: namely, wet market broiler versus backyard poultry and urban wet market wastewater versus rural wastewater.

The primary separation of samples by source is not surprising. Existing resistome surveys illustrate that different sample origins harbor distinct ARG assemblages (39, 40). In certain cases, these differences may reflect the microbial taxa which are adapted to specific environments and their intrinsic or commonly acquired resistance genes. For example, the *cfxA* beta-lactamase gene family has been identified as a dominant member of mammalian gut and fecal resistomes, including healthy humans (41), cattle (42, 43), and pigs (39, 44). Gatica et al. (40) also showed the robust association of *cfxA* genes with bovine and human fecal samples, while demonstrating their comparative scarcity in environmental samples.

The *cfxA* genes are well documented within the phylum Bacteroidetes, of which genera such as *Prevotella* spp. and *Bacteroides* spp. are abundant within mammalian anaerobic niches: the gastrointestinal tract and oral cavity. Furthermore, Suriyaphol et al. (45) found a positive correlation between *Prevotella* spp. and *cfxA6* in pig gut microbiomes. We found a positive correlation between *cfxA6* and *Prevotella* spp. across all samples in the current work. Our data therefore show that the dominant ARG group in human fecal samples corresponds well with the dominant bacterial genus within the same sample source (Supplementary Fig. S1 and Fig. S8).

However, in many cases, it was not possible to define such clear associations between highly abundant ARGs and bacterial genera in wastewater and poultry samples. For example, contigs harboring tetracycline genes such as *tetQ* and *tetW* could not be linked to specific taxa below the rank of order (Bacteroidales) or phylum (Firmicutes) in any sample origin. This likely corresponds to the decoupling of these genes from strict phylogenetic constraints by virtue of horizontal gene transfer. Indeed, *tetW* is known to have an extensive host range encompassing both Gram-positive and negative bacteria and can be integrated on conjugal transposons (46). The mobility of *tetQ* is less well documented, although it has been found on plasmids in *Bifidobacterium* strains (47).

Wastewater samples had the highest ARG subtype richness (^0^ *D*) compared with human and poultry samples consistent with having the greatest multivariate dispersion. Furthermore, Hill number extrapolations indicate the true richness of some wastewater samples may be underestimated due to under-sequencing. These findings are consistent with wastewater receiving varied inflow material, including washing detergents, cooking residues, human and animal waste, and residues from animal slaughter. The wastewater resistome is therefore not simply a combination of human and poultry ARGs. *FEAST* source attribution also supports this assertion, since less than half of the wastewater resistome in urban wet markets could be explained by the corresponding human and poultry samples.

The complexity and variability of the wastewater resistome are also likely to explain why several ARG categories discriminate wastewater from human and poultry samples (shown in Fig. 1). Another contributing factor could be co-localization of genes conferring resistance to different categories of antibiotic compounds. Several contigs assembled from wastewater samples support this notion: aminoglycoside resistance genes were frequently identified alongside beta-lactam, phenicol, trimethoprim, and fluoroquinolone resistance genes. Our results show examples of these contigs share homology with both the chromosomes of *Aeromona*s spp. and plasmids associated with *Enterobacteriaceae*.

The overall predominance of tetracycline resistance genes in the present study parallels a previous study showing these genes were prevalent in humans, goats, and chicken feces in Bangladesh (48). Tetracycline resistance genes have also been shown to dominate the resistomes of human, pig, and poultry feces in Chinese wet markets (49). Metagenomic studies on wastewater outlets in Bangladesh are limited, with most wastewater surveillance studies relying on cultivating select target organisms (15). To our knowledge, existing metagenomic studies on water resistomes in Bangladesh only include surface water (50). Since the wastewater outlets surveyed in the present work discharge directly into surface water, we discuss whether similar resistance genes were recovered in these compartments. McInnes et al. (50) found differences in the resistome of surface water and sediment obtained from rural (Mymensingh, Shariatpur) and urban (Dhaka) sites in Bangladesh. Specifically, the authors showed that urban surface waters were most enriched with macrolide, sulfonamide, aminoglycoside, and multidrug efflux resistance genes, relative to rural settings. In the current work, sulfonamide, aminoglycoside, and multidrug resistance genes were also among those prevalent in wastewater samples from urban wet markets (Fig. 1). McInnes et al. (50) suggested that human gut bacteria drive antibiotic resistance genes in surface water. Given surface water bodies in urban areas receive wastewater runoff from wet markets and mainstream sewage, both human and animal waste should be considered potential sources of these genes in the environment. Lastly, McInnes et al. (50) found tetracycline resistance genes made a comparatively minor contribution to surface water bodies in urban areas, indicating that while they dominate wastewater in our study, bacteria carrying these genes (though apparently diverse) may be less capable of competing in the wider aquatic environment.

Our findings suggest there was no significant difference between the abundance of tetracycline resistance genes in broiler poultry and backyard chicken ceca (Fig. 3). This was unexpected given that tetracycline is frequently used in commercial poultry farming (7, 51). Our household survey indicated that none of the rural poultry had received tetracycline antibiotics in the 4 weeks prior to sampling (7). Though vendors reported that they do not give antibiotics to broiler poultry at the point of sale, the small-scale commercial farms which supply bird markets regularly use antibiotics throughout the production cycle. In a survey of small-scale commercial broiler farmers, 65% reported using tetracyclines (7) with no withdrawal period before supplying birds to retail markets (8). It has been shown that tetracyclines can have long half-lives in the environment compared with other commonly used antibiotics (43), although many factors influence their persistence (52). The high prevalence of tetracycline resistance in both rural backyard chicken and broiler poultry may therefore indicate widespread contamination of the terrestrial environment with tetracycline residues and the proliferation of resistant bacteria.

A salient finding of the present work is that the resistomes of rural backyard chicken ceca are distinct from those of commercially reared poultry sold in urban wet markets. This appears to be driven in part by MLS and streptothricin (nucleoside) resistance genes, which were significantly enriched in wet market broilers relative to rural backyard chickens. It is likely that the higher stocking density, range, and frequency of antibiotic exposure in commercial broiler farms contribute to these differences. However, to test the impact of antibiotic use specifically, it would be necessary to establish a control group of birds raised without antibiotics under intensive and non-intensive conditions.

Although the wet market environment itself may play a role in shaping the resistome of broiler poultry, it is important to note that the surveyed birds spent less than 24 hours in the urban wet market prior to slaughter. Consequently, we contend that the rearing environment exerts a greater influence on the resistome of these animals. According to ordinations, the cecal resistome of rural backyard chickens was less distant from human resistomes than urban wet market broiler resistome compositions (Supplementary Fig. S2 and Fig. S3). This finding likely corresponds with microbiome sharing facilitated through routine direct contact with owners and indirect exchange through mutual exposure within household settings.

MLS-bearing contigs indicated multiple MLS resistance gene families were responsible for category-level enrichment in broiler poultry, with putative hosts among Firmicutes, Bacteroidetes, and Proteobacteria. MLS genes were occasionally co-localized with tetracycline, aminoglycoside, and beta-lactam resistance genes in various configurations. These included association with transposable elements, highlighting their mobility. Macrolide antibiotics are known to be widely marketed in Bangladesh (53) for both human and animal use; they have also been used by farms supplying urban wet markets surveyed in this study. Streptothricin, on the other hand, has only been used outside human clinical practice for prophylactic growth promotion in animals (54). We found no evidence of its use within poultry operations in Bangladesh. Broiler poultry contigs containing streptothricin resistance *sat*-family genes were commonly co-localized with aminoglycoside resistance genes and distributed across phyla (Firmicutes and Proteobacteria).

To date, few studies have applied metagenomic techniques to examine cecal resistomes in poultry samples, in Bangladesh or elsewhere. One study used a qPCR array to compare the fecal resistome of small-scale broiler chickens with backyard poultry in Ecuador (55). The authors found ARG richness in production chickens was significantly higher than that of household chickens; our metagenomic data indicated only a trend in Bangladesh (Kruskal-Wallis test; *χ*² = 6.5, df = 1, *P*_adj._ = 0.06312).

A recent publication by Swarthout et al. (48) used long-read sequencing to compare fecal resistomes of humans, goats, and chickens in urban and rural households in Bangladesh. There are several experimental and methodological differences between the aforementioned study and our current work. Firstly, only household backyard poultry (rural and urban) were sampled, whereas the current study sampled commercially reared broilers sold in urban wet markets and backyard poultry in rural households. Secondly, Swarthout et al. (48) separated urban and rural samples and then pooled DNA extractions, limiting the ability to discern the level of variability between individuals within these two groups. Thirdly, the current work obtained samples from poultry ceca whereas Swarthout et al. (48) sampled poultry feces collected from the environment.

Despite these methodological differences, some high-level findings correspond across both studies. For example, Swarthout et al. (48) did not report a significant difference between human fecal samples derived from urban and rural locations, which parallels our findings. However, Swarthout et al. (48) did not specifically survey urban wet market workers. This is significant, since our work extends previous findings, implying that the resistome of urban wet market workers is not dramatically altered by regular occupational exposure to broiler poultry viscera. Another study collected fecal samples from broiler farm chickens, live poultry market workers, and humans with low exposure to poultry in China (56). Their findings indicated that humans with low exposure to live poultry markets had significantly lower ARG diversity than live poultry market workers. Although we did not find significant differences between low- and high-exposure human fecal samples, this may relate to the widespread practice of keeping backyard chickens and/or general environmental contamination with antibiotic-resistant organisms in Bangladesh.

Although the total load and richness of ARGs are an important consideration when identifying potential areas to focus mitigation measures for AMR, specific combinations of ARGs and bacteria can present an immediate threat to human health, reflected in the WHO prioritized surveillance list (32).

We found GES carbapenemases in both rural and urban wastewater. These genes are of particular interest as the contigs suggested possible carriage on *Enterobacteriaceae* plasmids and *Pseudomonas aeruginosa* chromosomes, both “critical concern” organisms. A recent meta-analysis indicates that GES-2 genes are among the most widely distributed carbapenemase genes in aquatic environments, including wastewater, freshwater, and sediment (57). Likewise, GES-5 is carried by bacteria abundant in aquatic environments (58). Although Lin et al. (57) shows limited association of GES-2 with humans, culture-based analyses have previously identified this variant in nosocomial outbreaks of ESBL-producing *Pseudomonas aeruginosa* (59).

More broadly, the presence of carbapenemases (GES and OXA) indicates that while the total load and richness of ARGs may be greater in urban wastewater, rural wastewater should not be discounted as a source of ARGs critical to One Health initiatives.

It is noteworthy that previous real-time PCR studies have shown considerable prevalence of CTX-M-1 ESBLs, and to a lesser extent NDM-1 CRE genes, in wastewater from urban markets, poultry farms, and rural households (15). Detection of these genes was limited across our metagenomic data set. This may be a consequence of insufficient sequencing depth and the lack of targeted amplification for these lower abundance genes. Focusing on contigs allows more confident definition of taxon-ARG associations and potential ESBL/carbapenemase activity (since the complete genes can be screened against variant databases).

An important limitation of the study is the small sample size from each source which might have an impact on the generalizability of the results. In addition, use of short read sequencing can lead to incomplete assembly and many fragmented contigs with partial genes present at contig ends, which can lead to underestimated variant prevalence across different sources. Therefore, further studies with larger sample sizes are recommended to validate and strengthen the conclusions presented here. Future studies may consider hybrid sequencing or employing culture-enriched metagenomics to better study these clinically relevant genes (60). Similarly, sequencing depth can also influence the recovery of bacterial community members and ARGs, and we cannot rule out that deeper sequencing may yield additional insight into the extent of resistome sharing across hosts. The use of methods to target specific ARGs of interest, such as quantitative PCR, may also provide insight into the sharing of genes across hosts and environments, albeit at the expense of scope.

### Conclusion

We provide in-depth contextualization of resistomes associated with human fecal, poultry cecal, and wastewater samples in Bangladesh. We demonstrate that the impact of environmental setting on the resistome can differ depending on sample origin. The resistome of fecal samples originating from humans with and without routine occupational exposure to poultry is not significantly different. However, broiler poultry from urban wet markets have a significantly higher abundance of MLS and streptothricin ARGs compared with rural backyard chickens. The ARG compositions of human fecal and poultry cecal samples are also distinct. Overall, wastewater samples have the highest ARG richness and were under-sampled in our campaign. Nonetheless, rural wastewater was identified as a source of “priority one” antibiotic-resistant organisms selected by the WHO, highlighting wastewater in both urban and rural settings is a concern for human and animal health in Bangladesh. Wastewater is an important, but poorly understood component of One Health studies on AMR in Bangladesh. Further studies using long-read/hybrid or culture-enriched sequencing of rural backyard and broiler poultry in farms and in urban wet markets would generate a more complete understanding of how the poultry-rearing practices of medium- and small-scale farms in Bangladesh determine the resistome as opposed to the contaminated environments where free-range chickens roam. Finally, deeper sequencing is likely to reveal that wastewater in Bangladesh contains an even greater variety of ARGs than identified in the present work.

## Data Availability

Genomic data are available from the European Nucleotide Archive, study accession PRJEB48068. Other data relating to the wider project are openly accessible at https://doi.org/10.5285/0239cdaf-deab-4151-8f68-715063eaea45 and https://doi.org/10.5285/dda6dd55-f955-4dd5-bc03-b07cc8548a3d. Supplemental figures and tables are available at Loughborough.

## References

[1] Bezabih YM , Sabiiti W , Alamneh E , Bezabih A , Peterson GM , Bezabhe WM , Roujeinikova A . 2021. The global prevalence and trend of human intestinal carriage of ESBL-producing Escherichia coli in the community. J Antimicrob Chemother 76:22–29. doi:10.1093/jac/dkaa399 33305801

[2] Maillard J-Y , Bloomfield SF , Courvalin P , Essack SY , Gandra S , Gerba CP , Rubino JR , Scott EA . 2020. Reducing antibiotic prescribing and addressing the global problem of antibiotic resistance by targeted hygiene in the home and everyday life settings: a position paper. Am J Infect Control 48:1090–1099. doi:10.1016/j.ajic.2020.04.011 32311380 PMC7165117

[3] Van Boeckel TP , Brower C , Gilbert M , Grenfell BT , Levin SA , Robinson TP , Teillant A , Laxminarayan R . 2015. Global trends in antimicrobial use in food animals. Proc Natl Acad Sci U S A 112:5649–5654. doi:10.1073/pnas.1503141112 25792457 PMC4426470

[4] Myers J , Hennessey M , Arnold J-C , McCubbin KD , Lembo T , Mateus A , Kitutu FE , Samanta I , Hutchinson E , Davis A , Mmbaga BT , Nasuwa F , Gautham M , Clarke SE . 2022. Crossover-use of human antibiotics in livestock in agricultural communities: a qualitative cross-country comparison between Uganda, Tanzania and India. Antibiotics (Basel) 11:1342. doi:10.3390/antibiotics11101342 36290000 PMC9598773

[5] Madec J-Y , Haenni M , Nordmann P , Poirel L . 2017. Extended-spectrum β-lactamase/AmpC- and carbapenemase-producing Enterobacteriaceae in animals: a threat for humans? Clin Microbiol Infect 23:826–833. doi:10.1016/j.cmi.2017.01.013 28143782

[6] Nguyen VT , Jamrozy D , Matamoros S , Carrique-Mas JJ , Ho HM , Thai QH , Nguyen TNM , Wagenaar JA , Thwaites G , Parkhill J , Schultsz C , Ngo TH . 2019. Limited contribution of non-intensive chicken farming to ESBL-producing Escherichia coli colonization in humans in Vietnam: an epidemiological and genomic analysis. J Antimicrob Chemother 74:561–570. doi:10.1093/jac/dky506 30629197 PMC6376849

[7] Rousham EK , Asaduzzaman M , Mozmader TIMAU , Amin MB , Rahman M , Hossain MI , Islam MR , Mahmud ZH , Unicomb L , Islam MA . 2021. Human colonization with extended-spectrum beta-lactamase-producing E. coli in relation to animal and environmental exposures in Bangladesh: an observational one health study. Environ Health Perspect 129:37001. doi:10.1289/EHP7670 33656920 PMC7929562

[8] Masud AA , Rousham EK , Islam MA , Alam M-U , Rahman M , Mamun AA , Sarker S , Asaduzzaman M , Unicomb L . 2020. Drivers of antibiotic use in poultry production in Bangladesh: dependencies and dynamics of a patron-client relationship. Front Vet Sci 7:78. doi:10.3389/fvets.2020.00078 32185184 PMC7058630

[9] Alam M-U , Rahman M Islam MA , Asaduzzaman M , Sarker S , Rousham E , Unicomb L . 2019. Human exposure to antimicrobial resistance from poultry production: assessing hygiene and waste-disposal practices in Bangladesh. Int J Hyg Environ Health 222:1068–1076. doi:10.1016/j.ijheh.2019.07.007 31331788

[10] Larsson DGJ , Andremont A , Bengtsson-Palme J , Brandt KK , de Roda Husman AM , Fagerstedt P , Fick J , Flach C-F , Gaze WH , Kuroda M , Kvint K , Laxminarayan R , Manaia CM , Nielsen KM , Plant L , Ploy M-C , Segovia C , Simonet P , Smalla K , Snape J , Topp E , van Hengel AJ , Verner-Jeffreys DW , Virta MPJ , Wellington EM , Wernersson A-S . 2018. Critical knowledge gaps and research needs related to the environmental dimensions of antibiotic resistance. Environment International 117:132–138. doi:10.1016/j.envint.2018.04.041 29747082

[11] Rousham EK , Unicomb L , Islam MA. 2018. Human, animal and environmental contributors to antibiotic resistance in low-resource settings: integrating behavioural, epidemiological and one health approaches. Proceedings of the Royal Society B: Biological Sciences 285:20180332.R 10.1098/rspb.2018.0332 PMC5904322 29643217

[12] Pruden A , Vikesland PJ , Davis BC , de Roda Husman AM . 2021. Seizing the moment: now is the time for integrated global surveillance of antimicrobial resistance in wastewater environments. Curr Opin Microbiol 64:91–99. doi:10.1016/j.mib.2021.09.013 34655936

[13] Prieto Riquelme MV , Garner E , Gupta S , Metch J , Zhu N , Blair MF , Arango-Argoty G , Maile-Moskowitz A , Li A-D , Flach C-F , Aga DS , Nambi IM , Larsson DGJ , Bürgmann H , Zhang T , Pruden A , Vikesland PJ . 2022. Demonstrating a comprehensive wastewater-based surveillance approach that differentiates globally sourced resistomes. Environ Sci Technol 56:14982–14993. doi:10.1021/acs.est.1c08673 35759608 PMC9631994

[14] Rousham E , Unicomb L , Wood P , Smith M , Asaduzzaman M , Islam MA . 2018. Spatial and temporal variation in the community prevalence of antibiotic resistance in Bangladesh: an integrated surveillance study protocol. BMJ Open 8:e023158. doi:10.1136/bmjopen-2018-023158 PMC5931287 29705771

[15] Asaduzzaman M , Rousham E , Unicomb L , Islam MR , Amin MB , Rahman M , Hossain MI , Mahmud ZH , Szegner M , Wood P , Islam MA . 2022. Spatiotemporal distribution of antimicrobial resistant organisms in different water environments in urban and rural settings of Bangladesh. Sci Total Environ 831:154890. doi:10.1016/j.scitotenv.2022.154890 35364179

[16] Bolger AM , Lohse M , Usadel B . 2014. Trimmomatic: a flexible trimmer for Illumina sequence data. Bioinformatics 30:2114–2120. doi:10.1093/bioinformatics/btu170 24695404 PMC4103590

[17] Langmead B , Salzberg SL . 2012. Fast Gapped-read alignment with bowtie 2. Nat Methods 9:357–359. doi:10.1038/nmeth.1923 22388286 PMC3322381

[18] Li D , Liu C-M , Luo R , Sadakane K , Lam T-W . 2015. MEGAHIT: an ultra-fast single-node solution for large and complex metagenomics assembly via succinct de Bruijn graph. Bioinformatics 31:1674–1676. doi:10.1093/bioinformatics/btv033 25609793

[19] Yin X , Jiang X-T , Chai B , Li L , Yang Y , Cole JR , Tiedje JM , Zhang T , Wren J . 2018. Args-OAP V2. 0 with an expanded SARG database and hidden markov models for enhancement characterization and quantification of antibiotic resistance genes in environmental metagenomes. Bioinformatics 34:2263–2270. doi:10.1093/bioinformatics/bty053 29408954

[20] Feng J , Li B , Jiang X , Yang Y , Wells GF , Zhang T , Li X . 2018. Antibiotic resistome in a large‐scale healthy human gut microbiota deciphered by metagenomic and network analyses. Environ Microbiol 20:355–368. doi:10.1111/1462-2920.14009 29194931

[21] Murray AK , Zhang L , Snape J , Gaze WH . 2019. Comparing the selective and co-selective effects of different antimicrobials in bacterial communities. Int J Antimicrob Agents 53:767–773. doi:10.1016/j.ijantimicag.2019.03.001 30885807 PMC6546120

[22] Qian X , Gunturu S , Guo J , Chai B , Cole JR , Gu J , Tiedje JM . 2021. Metagenomic analysis reveals the shared and distinct features of the soil resistome across tundra, temperate prairie, and tropical ecosystems. Microbiome 9:1–13. doi:10.1186/s40168-021-01047-4 33990222 PMC8122544

[23] Buchfink B , Reuter K , Drost H-G . 2021. Sensitive protein alignments at tree-of-life scale using DIAMOND. Nat Methods 18:366–368. doi:10.1038/s41592-021-01101-x 33828273 PMC8026399

[24] Alcock BP , Raphenya AR , Lau TTY , Tsang KK , Bouchard M , Edalatmand A , Huynh W , Nguyen A-L , Cheng AA , Liu S , Min SY , Miroshnichenko A , Tran H-K , Werfalli RE , Nasir JA , Oloni M , Speicher DJ , Florescu A , Singh B , Faltyn M , Hernandez-Koutoucheva A , Sharma AN , Bordeleau E , Pawlowski AC , Zubyk HL , Dooley D , Griffiths E , Maguire F , Winsor GL , Beiko RG , Brinkman FSL , Hsiao WWL , Domselaar GV , McArthur AG . 2020. CARD 2020: antibiotic resistome surveillance with the comprehensive antibiotic resistance database. Nucleic Acids Res 48:D517–D525. doi:10.1093/nar/gkz935 31665441 PMC7145624

[25] Menzel P , Ng KL , Krogh A . 2016. Fast and sensitive taxonomic classification for metagenomics with Kaiju. Nat Commun 7:11257. doi:10.1038/ncomms11257 27071849 PMC4833860

[26] Sayers EW , Agarwala R , Bolton EE , Brister JR , Canese K , Clark K , Connor R , Fiorini N , Funk K , Hefferon T , Holmes JB , Kim S , Kimchi A , Kitts PA , Lathrop S , Lu Z , Madden TL , Marchler-Bauer A , Phan L , Schneider VA , Schoch CL , Pruitt KD , Ostell J . 2019. Database resources of the national center for biotechnology information. Nucleic Acids Res 47:D23–D28. doi:10.1093/nar/gky1069 30395293 PMC6323993

[27] Morgulis A , Coulouris G , Raytselis Y , Madden TL , Agarwala R , Schäffer AA . 2008. Database indexing for production megablast searches. Bioinformatics 24:1757–1764. doi:10.1093/bioinformatics/btn322 18567917 PMC2696921

[28] Clarke K , Gorley R . 2006. Primer. PRIMER-e, Plymouth 866

[29] Anderson M , Gorley R , Clarke K. 2008. PERMANOVA+ for PRIMER: guide to software and statistical methods, PRIMER-E Ltd.

[30] Hsieh TC , Ma KH , Chao A , McInerny G . 2016. iNEXT: an R package for rarefaction and extrapolation of species diversity (H ill numbers) . Methods Ecol Evol 7:1451–1456. doi:10.1111/2041-210X.12613

[31] Leo Lahti SS. 2017. Tools for microbiome analysis in R. Microbiome package. http://microbiome.github.com/microbiome.

[32] WHO . 2017. Global priority list of antibiotic-resistant bacteria to guide research, discovery, and development of new antibiotics

[33] Legendre P . 2018. lmodel2 R package.

[34] Shenhav L , Thompson M , Joseph TA , Briscoe L , Furman O , Bogumil D , Mizrahi I , Pe’er I , Halperin E . 2019. FEAST: fast expectation-maximization for microbial source tracking. Nat Methods 16:627–632. doi:10.1038/s41592-019-0431-x 31182859 PMC8535041

[35] Chen J , Chen H , Liu C , Huan H , Teng Y . 2023. Evaluation of FEAST for metagenomics-based source tracking of antibiotic resistance genes. J Hazard Mater 442:130116. doi:10.1016/j.jhazmat.2022.130116 36209606

[36] Bonnin RA , Jousset AB , Emeraud C , Oueslati S , Dortet L , Naas T . 2020. Genetic diversity, biochemical properties, and detection methods of minor carbapenemases in enterobacterales. Front Med (Lausanne) 7:616490. doi:10.3389/fmed.2020.616490 33553210 PMC7855592

[37] Evans BA , Hamouda A , Towner KJ , Amyes SGB . 2008. OXA-51-like beta-Lactamases and their association with particular epidemic lineages of Acinetobacter baumannii. Clin Microbiol Infect 14:268–275. doi:10.1111/j.1469-0691.2007.01919.x 18190566

[38] Poirel L , Marqué S , Héritier C , Segonds C , Chabanon G , Nordmann P . 2005. OXA-58, a novel class D β-lactamase involved in resistance to carbapenems in Acinetobacter baumannii . Antimicrob Agents Chemother 49:202–208. doi:10.1128/AAC.49.1.202-208.2005 15616297 PMC538857

[39] Li B , Yang Y , Ma L , Ju F , Guo F , Tiedje JM , Zhang T . 2015. Metagenomic and network analysis reveal wide distribution and co-occurrence of environmental antibiotic resistance genes. ISME J 9:2490–2502. doi:10.1038/ismej.2015.59 25918831 PMC4611512

[40] Gatica J , Jurkevitch E , Cytryn E . 2019. Comparative metagenomics and network analyses provide novel insights into the scope and distribution of β-lactamase homologs in the environment. Front Microbiol 10:146. doi:10.3389/fmicb.2019.00146 30804916 PMC6378392

[41] Hu Y , Yang X , Qin J , Lu N , Cheng G , Wu N , Pan Y , Li J , Zhu L , Wang X , Meng Z , Zhao F , Liu D , Ma J , Qin N , Xiang C , Xiao Y , Li L , Yang H , Wang J , Yang R , Gao GF , Wang J , Zhu B . 2013. Metagenome-wide analysis of antibiotic resistance genes in a large cohort of human gut microbiota. Nat Commun 4:1–7. doi:10.1038/ncomms3151 23877117

[42] Zhou B , Wang C , Zhao Q , Wang Y , Huo M , Wang J , Wang S . 2016. Prevalence and dissemination of antibiotic resistance genes and coselection of heavy metals in Chinese dairy farms. J Hazard Mater 320:10–17. doi:10.1016/j.jhazmat.2016.08.007 27505289

[43] Baker M , Williams AD , Hooton SPT , Helliwell R , King E , Dodsworth T , María Baena-Nogueras R , Warry A , Ortori CA , Todman H , Gray-Hammerton CJ , Pritchard ACW , Iles E , Cook R , Emes RD , Jones MA , Kypraios T , West H , Barrett DA , Ramsden SJ , Gomes RL , Hudson C , Millard AD , Raman S , Morris C , Dodd CER , Kreft J-U , Hobman JL , Stekel DJ . 2022. Antimicrobial resistance in dairy slurry tanks: a critical point for measurement and control. Environ Int 169:107516. doi:10.1016/j.envint.2022.107516 36122459

[44] Lim S-K , Kim D , Moon D-C , Cho Y , Rho M . 2020. Antibiotic resistomes discovered in the gut microbiomes of Korean swine and cattle. Gigascience 9:giaa043. doi:10.1093/gigascience/giaa043 32369165 PMC7317084

[45] Suriyaphol P , Chiu JKH , Yimpring N , Tunsagool P , Mhuantong W , Chuanchuen R , Bessarab I , Williams RBH , Ong RT-H , Suriyaphol G . 2021. Dynamics of the fecal microbiome and antimicrobial resistome in commercial piglets during the weaning period. Sci Rep 11:18091. doi:10.1038/s41598-021-97586-9 34508122 PMC8433359

[46] Roberts MC . 2005. Update on acquired tetracycline resistance genes. FEMS Microbiol Lett 245:195–203. doi:10.1016/j.femsle.2005.02.034 15837373

[47] Ma Y , Xie T-T , Hu Q , Qiu Z , Song F . 2015. Sequencing analysis and characterization of the plasmid pBIF10 isolated from Bifidobacterium Longum. Can J Microbiol 61:124–130. doi:10.1139/cjm-2014-0581 25587774

[48] Swarthout JM , Fuhrmeister ER , Hamzah L , Harris AR , Ahmed MA , Gurley ES , Satter SM , Boehm AB , Pickering AJ . 2022. Differential overlap in human and animal fecal microbiomes and resistomes in rural versus urban Bangladesh. Appl Environ Microbiol 88:e0075922. doi:10.1128/aem.00759-22 35862660 PMC9317872

[49] Wang Y , Hu Y , Cao J , Bi Y , Lv N , Liu F , Liang S , Shi Y , Jiao X , Gao GF , Zhu B . 2019. Antibiotic resistance gene reservoir in live poultry markets. J Infect 78:445–453. doi:10.1016/j.jinf.2019.03.012 30935879

[50] McInnes RS , Uz-Zaman MH , Alam IT , Ho SFS , Moran RA , Clemens JD , Islam MS , van Schaik W . 2021. Metagenome-wide analysis of rural and urban surface waters and sediments in Bangladesh identifies human waste as a driver of antibiotic resistance. mSystems 6:e0013721. doi:10.1128/mSystems.00137-21 34254820 PMC8407206

[51] Hasan B , Faruque R , Drobni M , Waldenström J , Sadique A , Ahmed KU , Islam Z , Parvez MBH , Olsen B , Alam M . 2011. High prevalence of antibiotic resistance in pathogenic Escherichia coli from large-and small-scale poultry farms in Bangladesh. Avian Dis. 55:689–692. doi:10.1637/9686-021411-Reg.1 22312993

[52] Cycoń M , Mrozik A , Piotrowska-Seget Z . 2019. Antibiotics in the soil environment—degradation and their impact on microbial activity and diversity. Front Microbiol 10:338. doi:10.3389/fmicb.2019.00338 30906284 PMC6418018

[53] Orubu ESF , Samad MA , Rahman MT , Zaman MH , Wirtz VJ . 2021. Mapping the antimicrobial supply chain in Bangladesh: a scoping-review-based ecological assessment approach. Glob Health Sci Pract 9:532–547. doi:10.9745/GHSP-D-20-00502 34593580 PMC8514039

[54] Webb HE , Angulo FJ , Granier SA , Scott HM , Loneragan GH . 2017. Illustrative examples of probable transfer of resistance determinants from food animals to humans: streptothricins, glycopeptides, and colistin. F1000Res 6:1805. doi:10.12688/f1000research.12777.1 29188021 PMC5686510

[55] Guo X , Stedtfeld RD , Hedman H , Eisenberg JNS , Trueba G , Yin D , Tiedje JM , Zhang L . 2018. Antibiotic resistome associated with small-scale poultry production in rural ecuador. Environ Sci Technol 52:8165–8172. doi:10.1021/acs.est.8b01667 29944836

[56] Wang Y , Lyu N , Liu F , Liu WJ , Bi Y , Zhang Z , Ma S , Cao J , Song X , Wang A , Zhang G , Hu Y , Zhu B , Gao GF . 2021. More diversified antibiotic resistance genes in chickens and workers of the live poultry markets. Environment International 153:106534. doi:10.1016/j.envint.2021.106534 33799229

[57] Lin Q , Xavier BB , Alako BTF , Mitchell AL , Rajakani SG , Glupczynski Y , Finn RD , Cochrane G , Malhotra-Kumar S . 2022. Screening of global microbiomes implies ecological boundaries Impacting the distribution and dissemination of clinically relevant antimicrobial resistance genes. Commun Biol 5:1217. doi:10.1038/s42003-022-04187-x 36400841 PMC9674584

[58] Manageiro V , Ferreira E , Caniça M , Manaia CM . 2014. GES-5 among the β-lactamases detected in ubiquitous bacteria isolated from aquatic environment samples. FEMS Microbiol Lett 351:64–69. doi:10.1111/1574-6968.12340 24267783

[59] Poirel L , Weldhagen GF , De Champs C , Nordmann P . 2002. A Nosocomial outbreak of Pseudomonas aeruginosa isolates expressing the extended-spectrum β-lactamase GES-2 in South Africa. J Antimicrob Chemother 49:561–565. doi:10.1093/jac/49.3.561 11864961

[60] Zhang Z , Zhang G , Ju F . 2022. Using culture-enriched phenotypic metagenomics for targeted high-throughput monitoring of the clinically important fraction of the β-lactam resistome. Environ Sci Technol 56:11429–11439. doi:10.1021/acs.est.2c03627 35930686

